# Self-reported cultural competency measures among patients with diabetes: A nationwide cross-sectional study in the United States

**DOI:** 10.1016/j.lana.2021.100158

**Published:** 2021-12-30

**Authors:** Joseph Alexander Paguio, Jem Marie Golbin, Jasper Seth Yao, Michelle Ann Eala, Edward Christopher Dee, Marc Gregory Yu

**Affiliations:** a Albert Einstein Medical Center, Philadelphia, PA, USA; b University of the Philippines College of Medicine, Manila, Philippines; c Memorial Sloan Kettering Cancer Center, NY, USA; d Harvard Medical School, Boston, MA, USA; e Joslin Diabetes Center, Boston, MA, USA

**Keywords:** Diabetes, Diabetes care, Access to healthcare, Culture, Cultural competency, Minorities, Diversity

## Abstract

**Background:**

Culturally tailored interventions may reduce disparities in diabetes care. We conducted a nationally representative assessment of self-reported cultural competency measures of care among patients with diabetes in the United States.

**Methods:**

The 2017 National Health Interview Survey was queried for adults with self-reported diabetes. Sample weight-adjusted multivariable logistic regressions defined adjusted odds ratios and 95% confidence intervals of a positive response to each of the cultural competency measures while controlling for relevant sociodemographic variables.

**Findings:**

2,448 adults were included in the analyses. Black, Latinx, and Asian respondents had greater odds of and individuals with the highest income level had lower odds of placing greater importance in sharing cultures with their provider. Black and Latinx individuals had lower odds of reporting encountering providers who shared or understood their cultures. Asians had lower odds of and respondents aged 40-64 and 65 years and older had greater odds of reporting frequently being treated with respect by their providers. Non-English speakers had lower odds of and individuals from higher income brackets had greater odds of reporting frequently receiving easy-to-understand information about their care. Blacks and respondents not part of the workforce had greater odds of reporting frequently being asked about their opinions or beliefs in care.

**Interpretation:**

Disparities in self-reported provider cultural competency measures exist among cultural minorities in the United States. Our findings may inform efforts to reduce disparities and improve care among minorities with diabetes.

**Funding:**

No funding was used in the preparation of this work.

**Research in Context**

**Evidence before this study**

A systematic review and prospective observational studies have demonstrated that culturally tailored interventions lead to improvements in glycemic control, cardiovascular risk factors, and dietary behaviors and reduction of disparities among racial minority groups with diabetes. In 2017, the American Diabetes Association gave a grade A recommendation for providers to assess the social context of their patients, which include ethnic/cultural/sex differences and language barriers, and to apply the information to treatment recommendations. Despite the promising benefits of culturally tailored strategies, a nationally representative assessment of self-reported provider cultural competency in diabetes care is lacking. Furthermore, most of the research in this field have pivoted on race and ethnicity without accounting for the other facets of culture.

**Added value of this study**

In this contemporary and nationally representative cross-sectional study of adults with diabetes in the United States, we found that while Black, Latinx, and Asian individuals placed greater importance on sharing cultures with their providers, Black and Latinx respondents were less likely to report encountering physicians who shared or understood their culture. Asian respondents were less likely to report being treated with respect. Non-English speakers were less likely to report receiving easy-to-understand information. These results reveal disparities in cultural competency measures in the care of patients with diabetes and offer an opportunity for future efforts to explore interventions that address these issues.

**Implication of all the available evidence**

Despite efforts to incorporate cultural competency in medical education and clinical practice, we have identified possible gaps in the care of cultural minorities who have diabetes in the United States. Our findings may inform efforts and policies concerning healthcare workforce representation, medical education, language assistive services, and health promotion. We hope this study encourages future assessments of cultural competency in healthcare to consider the other aspects of patients’ cultures, such as religion, sexual orientation, gender identity and expression (SOGIE), urban-rural household classification, languages spoken, and country of origin.

Alt-text: Unlabelled box

## Introduction

In the past year, there has been increased awareness about social inequities that lead to poorer health outcomes among racial and ethnic minorities, people of low socioeconomic status (SES), people who identify as lesbian, gay, bisexual, transgender, and queer/questioning (LGBTQ), and immigrants.^1^, ^2^, ^3^ The coronavirus disease 2019 (COVID-19) pandemic, and the Black Lives Matter and Stop Asian Hate movements have drawn attention to the consequences of longstanding structural racism and discrimination and the lack of investment in social and health services of at-risk populations.^4^^,^^5^

In the realm of diabetes, these healthcare disparities are well-documented.^6^ In 2017, the American Diabetes Association (ADA) identified populations at-risk for diabetes and worse outcomes.^6^ Racial and ethnic minorities, women, uninsured or underinsured patients, homeless individuals, and non-English speakers were at an increased risk for a range of complications in diabetes care, such as poor glycemic control, inadequate blood glucose monitoring, coronary heart disease, food insecurity, and hypoglycaemia.^6^ Due to the chronic nature of diabetes care and reliance on patient self-management, tailoring treatment to the social and cultural contexts of patients is crucial to improve adherence to the recommended standards.^6^

Provision of culturally competent care is one of the ways to address gaps in the care of culturally and linguistically diverse (CALD) populations.^7^ Past studies have demonstrated improvements in glycaemic control, cardiovascular risk factors, and dietary behaviors and reduction of disparities among populations with diabetes who received culturally tailored interventions.^6^^,^^8^, ^9^, ^10^ However, the majority of these studies stratified patient populations by race or ethnicity with less emphasis on other determinants of health disparities such as sexual orientation, household income, educational attainment, employment, migration status, and others. Interventions designed to increase the cultural competency of physicians have an unclear impact on patient outcomes such as glycaemic control or blood pressure control.^11^ Moreover, a contemporary and nationally representative assessment of physician cultural competency and patient preferences for cultural competency in diabetes care is lacking. We therefore examined self-reported cultural competency measures among patients with diabetes in the United States.

## Methods

The National Health Interview Survey (NHIS) collects health indicators from non-institutionalised, civilian adults in the United States. The database was queried for respondents from the year 2017–the only year during which questions related to cultural competency were explicitly asked–and those with a self-reported history of diabetes and complete sociodemographic data were included in the analyses. Self-reported diabetes was determined among adult respondents who answered “yes” to the question, "Have you ever been told by a doctor or health professional that you have diabetes or sugar diabetes?". Among female respondents, the question was preceded by a phrase, “Other than during pregnancy”, to exclude cases of gestational diabetes.

In 2017, the Office of Minority Health (OMH) funded a set of supplemental questions in the NHIS to collect nationally representative data on the perceived frequency of access to and importance of culturally competent care based on the National Standards for Culturally and Linguistically Appropriate Services (CLAS) in Health and Health Care.^12^ These questions were (1) “Some people think it is important for their providers to understand or share their race or ethnicity or gender or religion or beliefs or native language. How important is it to you that your health care providers understand or are similar to you in any of these ways?”. Those who answered slightly, somewhat or very important in this question were asked a follow up question, (2) “Some people think it is important for their providers to understand or share their race or ethnicity or gender or religion or beliefs or native language. How often were you able to see health care providers who were similar to you in any of these ways?”. The rest of the supplemental questions were (3) “How often were you treated with respect by your health care providers, (4) “How often did your health care providers tell or give you information about your health and health care that was easy to understand?”, (5) “How often did your health care providers ask for your opinions or beliefs about your medical care or treatment? For example, what kind of tests, procedures, or medications you prefer.” This supplemental set of questions was administered only to respondents who indicated that they had visited a physician/healthcare provider in the past year.

The dependent variable of interest was the response to each of these questions, which were binarised a priori as “very/somewhat important” vs. “slightly/not at all important” for survey question number 1 and “always/most of the time” vs. “some of the time/none of the time” for survey questions 2 to 5.

Survey weights, which account for the complex sampling methods of the NHIS, were used to produce nationally representative data. All analyses incorporated weights using the Stata *–svy–* command for structured survey data. Sample weight-adjusted multivariable logistic regressions defined adjusted odds ratios (aOR) and 95% confidence intervals (95%CI) of a positive response to each of the cultural competency outcomes while controlling for sociodemographic and clinical variables considered relevant to diabetes outcomes, such as age, sex, race and ethnicity, sexual orientation, insurance status, household income, educational attainment, employment status, and the ability to speak the English language (Table 1).^6^ These variables were chosen because of their associations with access to diabetes care; for example, insurance, race, and socioeconomic status are associated with disparities in access to quality diabetes care.^13^ Additionally, considering that culture can be conceptualised as “integrated patterns of human behavior that include the language, thoughts, communications, actions, customs, beliefs, values, and institutions of racial, ethnic, religious, or social groups,”^14^ variables such as sexual identity and language were also included. Of note, age groups were defined as 18–39, 40–64, and 65 and up, in line with prior NHIS studies, with the youngest cohort as the referent value given the ordinal nature of age groups.^15^ Only respondents with complete sociodemographic data who responded to all questions were included. Those with missing data or whose answers were “unknown” were excluded from analyses.

**Table 1:** Baseline demographic and clinical characteristics of adults with self-reported history of diabetes who responded to at least one of the cultural competency survey questions of the National Health Interview Survey (NHIS).

	*n*	Unweighted proportion (%)	Sample-weighted proportion (%)
	2448	100%	100%
Age, years			
18–39	163	6.66	8.44
40–64	1107	45.22	51.27
65 and over	1178	48.12	40.29
Female sex	1237	50.53	47.56
Race			
Non-Hispanic (NH) White	1617	66.05	61.14
NH Black	350	14.30	13.67
Hispanic	295	12.05	16.58
Asian	102	4.17	5.53
Other	84	3.43	3.09
Non-straight sexual orientation	67	2.74	2.32
Non-insured status	123	5.02	6.10
Non-US mainland-born (foreign-born)	288	11.76	16.5
Non-English speaker	83	3.39	5.14
Educational attainment			
Grade 8	161	6.58	7.61
Grade 12, no diploma	261	10.66	10.67
High school diploma	679	27.74	27.34
Some college	788	32.19	31.16
Bachelor's degree	329	13.44	13.84
Advanced degree	230	9.40	9.37
Employment status			
Employed	858	35.05	38.87
Unemployed	47	1.92	2.42
Not in workforce/retired	1543	63.03	58.71
Household income/poverty threshold*			
<1.00	378	15.44	13.56
1.00-1.99	579	23.65	22.97
2.00 or greater	1491	60.91	63.47

⁎ Ratio of household income to the poverty threshold.

Statistical analyses were conducted using STATA/IC 16.1 (StataCorp) with α=0.05. This study was deemed exempt from institutional review due to the secondary analyses of publicly available and de-identified data. Verbal consent was obtained by interviewers from all respondents before participation.^16^

### Role of the funding source

No funding was involved in this research.

## Results

The adult response rate in the year 2017 was 80.7%, in which there were 26,742 completed interviews out of 33,143 eligible sample adults.^16^ From this sample, 2812 adult respondents had self-reported diabetes, of whom 2448 had complete sociodemographic data and were included in the analyses. After weighting, 47.6% of the study population were female, 61.1% were White, 13.7% were Black, 16.6% were Latinx, 5.5% were Asian, 5.1% were non-English speakers, and 16.5% were foreign-born. The median age was 64 years (interquartile range 54–72). Sociodemographic characteristics of adults with diabetes who responded to at least one of the cultural competency survey questions are in Table 1. There were 1177 respondents (48.1%) who reported that it was at least slightly important that their providers shared/understood their cultures; these sample respondents were subsequently asked how frequently they encountered such providers.

**Figure 1:**
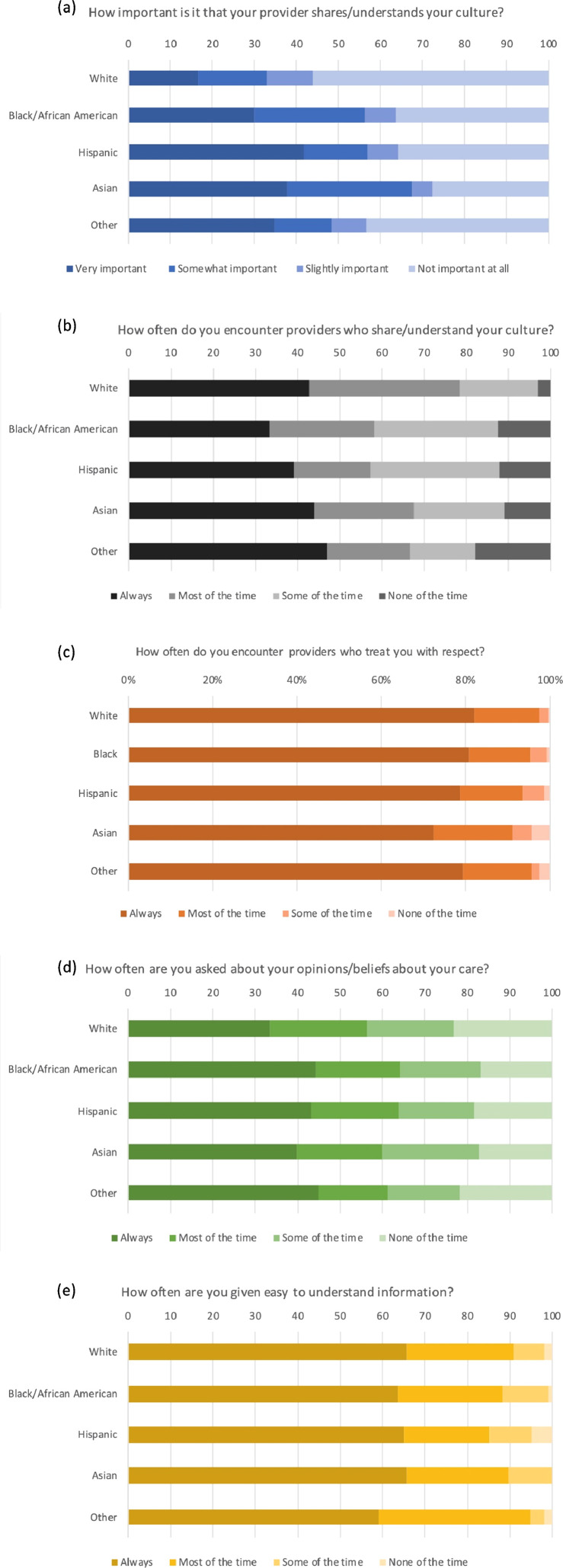
Weighted percentage of responses to patient-reported cultural competency measures of care among adults in the US in the year 2017, by race/ethnicity.

The odds of reporting that it was very important or somewhat important that their providers understood or shared their cultures were greater among Black, Latinx, and Asian respondents (Black aOR 2.16 95%CI [1.60–2.92], *P* < 0.001; Latinx aOR 1.63 95%CI [1.13–2.35], *P* = 0.01; Asian aOR 3.03, 95% CI[1.69–5.42], *P* < 0.001) compared to those of White respondents (Fig. 1). Compared to US-born respondents, foreign-born individuals had 1.41 (aOR, 95%CI [0.94–2.13]) times greater odds of reporting that it was very/somewhat important to them that providers shared or understood their culture, but this finding was not statistically significant (*P* = 0.10). Individuals whose household income was at least 2.00 times the poverty threshold had lower odds of reporting that it was important their providers share their culture (aOR 0.64, 95%CI [0.46–0.89], *P* = 0.01, Table 2 for all) compared to individuals whose household income was below the poverty threshold.

**Table 2:** Sample-weight adjusted multivariable logistic regressions defined adjusted odds ratios (aOR) of a positive response to each of the five cultural competency measures as the independent variable of interest. In survey questions 1 to 4, participants selected four possible answers: “always,” “most of the time,” “some of the time,” or “none of the time.” Answers were binarized a priori as “always/most of the time” vs. “some of the time/none of the time”. In survey question 5, participants selected four possible answers: “very important,” “somewhat important,” “slightly important,” or “not important at all.” Answers were binarized a priori as “very/somewhat important” vs. “slightly/not at all important”.

	How often do you see HCPs who were similar to you in culture?				How often were you treated with respect by your HCPs?				How often did you HCPs give you information that was easy to understand?			
	aOR	95%CI		*P*	aOR	95%CI		*P*	aOR	95%CI		*P*
Age, years Refs. 18, 19, 20, 21, 22, 23, 24, 25, 26, 27, 28, 29, 30, 31, 32, 33, 34, 35, 36, 37, 38, 39												
40–64	1.04	0.54	2.00	0.91	2.84	1.13	7.12	0.03	1.44	0.69	2.98	0.33
65 and over	1.32	0.63	2.78	0.46	6.32	2.22	18.03	0.001	1.41	0.64	3.09	0.40
Female sex (ref. male)	0.87	0.62	1.23	0.44	0.78	0.42	1.43	0.42	1.19	0.80	1.75	0.39
Race (ref. non-Hispanic white)												
NH Black	0.38	0.24	0.58	<0.001	0.74	0.38	1.46	0.40	0.89	0.55	1.47	0.66
Hispanic	0.37	0.22	0.65	<0.001	0.41	0.16	1.07	0.07	1.02	0.50	2.06	0.96
Asian	0.68	0.30	1.54	0.35	0.20	0.07	0.58	0.003	1.23	0.42	3.57	0.71
Others	0.54	0.23	1.27	0.16	0.81	0.22	2.94	0.75	1.92	0.64	5.74	0.24
Non-straight sexual orientation (ref. straight)	0.42	0.16	1.15	0.09	2.50	0.57	10.90	0.22	3.37	0.75	15.07	0.11
Non-insured status (vs. insured)	1.04	0.53	2.06	0.90	1.81	0.65	5.03	0.26	1.37	0.64	2.92	0.41
Nativity (ref. US mainland-born)												
Foreign-born	0.79	0.43	1.46	0.46	1.61	0.59	4.34	0.35	0.75	0.36	1.58	0.45
Non-English speaker (ref. English speaker)	0.96	0.42	2.20	0.93	1.32	0.44	4.02	0.62	0.39	0.17	0.90	0.03
Educational attainment (ref. grade 8)												
Grade 12, no diploma	0.71	0.32	1.57	0.40	0.61	0.19	1.97	0.41	0.90	0.42	1.90	0.77
High school diploma	0.92	0.45	1.89	0.82	1.15	0.38	3.45	0.80	0.68	0.33	1.43	0.31
Some college	0.96	0.44	2.06	0.91	1.49	0.50	4.41	0.47	0.81	0.38	1.73	0.59
Bachelor's degree	0.86	0.39	1.89	0.71	1.82	0.50	6.62	0.36	1.24	0.50	3.12	0.64
Advanced degree	0.75	0.32	1.74	0.50	2.15	0.52	8.84	0.29	2.12	0.78	5.75	0.14
Employment status (ref. employed)												
Unemployed	1.14	0.36	3.54	0.83	1.56	0.21	11.51	0.66	1.54	0.42	5.66	0.51
Not part of the workforce	0.97	0.62	1.51	0.89	0.62	0.32	1.19	0.15	1.38	0.85	2.22	0.19
Socioeconomic status *(ref. <1.00)												
1.00-1.99	0.82	0.48	1.41	0.48	1.78	0.87	3.64	0.12	1.96	1.16	3.31	0.01
2.00 or greater	0.66	0.39	1.14	0.13	1.75	0.86	3.56	0.12	2.34	1.43	3.83	0.001

⁎ Ratio of household income to the poverty threshold.

Black and Latinx individuals had decreased odds of reporting frequently encountering providers who shared or understood their cultures (Black aOR 0.38, 95%CI [0.24–0.58], *P* < 0.001; Latinx aOR 0.37, 95%CI [0.22–0.65], *P* < 0.001) compared to White respondents (Fig. 1). Compared to straight respondents, LGBTQ adults had 0.42 (aOR, 95%CI [0.16–1.15]) times lower odds of reporting frequently encountering providers who shared or understood their culture, but this finding was not statistically significant (*P* = 0.09, Table 2 for all).

Asians had lower odds of reporting frequently being treated with respect by their providers (aOR 0.20, 95%CI [0.07–0.58], *P* = 0.003) compared to White respondents (Fig. 1). Latinx respondents had 0.41 (aOR, 95%CI [0.16–1.07]) times lower odds of reporting being treated with respect by their providers, but this finding was not statistically significant (*P* = 0.07) (Fig. 1). Compared to adults aged 18–39 years, older age groups had greater odds of reporting being treated with respect by their providers (40–64 years aOR 2.84, 95%CI [1.13–7.12], *P* = 0.03; 65 years and older aOR 6.32, 95%CI [2.22–18.03], *P* = 0.001, Table 2 for all).

**Figure 2:**
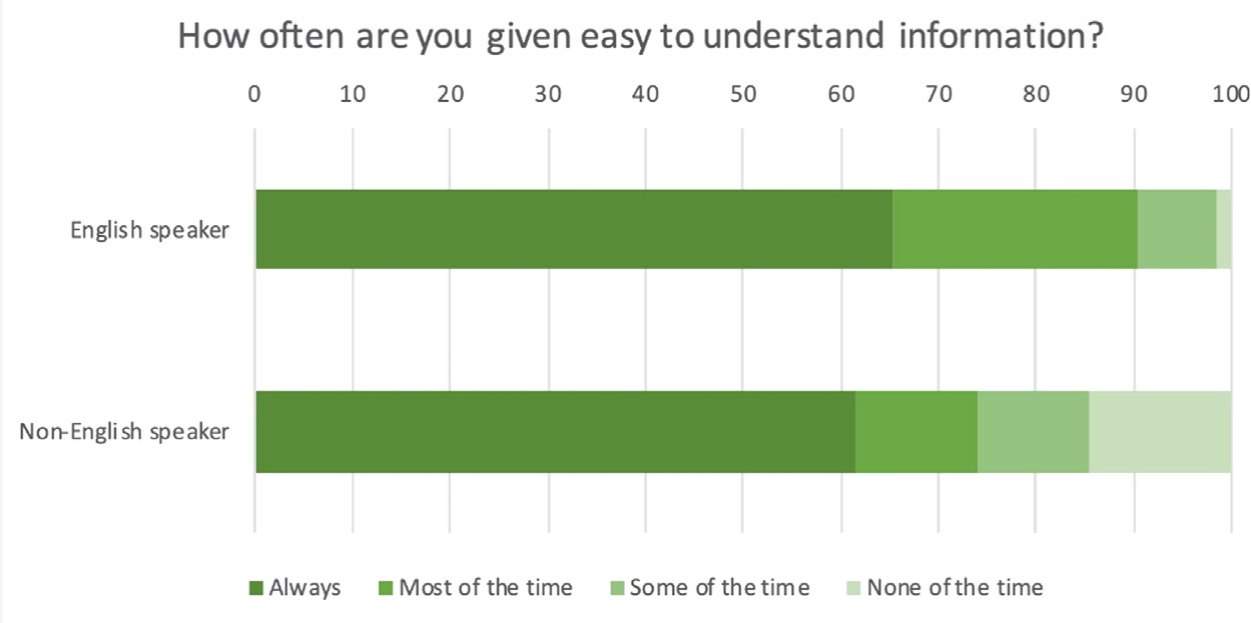
Weighted percentage of responses to self-reported frequency of receiving easy-to-understand information among adults with diabetes in the US in 2017, by use of the English language.

Compared to respondents who speak the English language, Non-English speakers had lower odds of reporting encountering physicians who provide easy-to-understand information (aOR 0.39, 95%CI [0.17 – 0.90], *P* = 0.03) (Fig. 2). Individuals whose household income was 1.00–1.99 and at least 2.00 times the poverty threshold had greater odds of reporting frequently receiving easy-to-understand information about their care (aOR 1.96, 95%CI [1.16–3.31], *P* = 0.01 & aOR 2.34, 95%CI [1.43–3.83], *P* = 0.001, respectively, Table 2 for all) compared to those whose household income was below the poverty threshold.

Blacks and adults not part of the workforce (e.g. retired) had greater odds of reporting frequently being asked about their opinions or beliefs about their care (Black aOR1.39, 95%CI [1.02–1.89], *P* = 0.036; workforce non-participation aOR 1.33, 95%CI [1.03–1.72], *P* = 0.030, Table 2 for all) compared to White and employed respondents, respectively (Fig. 1).

## Discussion

### Cultural competency and workforce diversity in health care

Our findings suggest that among adult respondents with diabetes, Black, Latinx, and Asian individuals place greater importance on sharing cultures with their providers, but Black and Latinx respondents less frequently encountered physicians who shared or understood their culture (Fig. 1). While point estimates demonstrate greater odds among foreign-born individuals to place greater importance on sharing cultures with providers and lower odds among LGBTQ adults to frequently encounter physicians who shared their culture, these findings may be underpowered due to the lack of an adequate sample size and the great heterogeneity among migrants and LGBTQ adults not captured in this study.

Racial and ethnic disparities in healthcare may be partly explained by the lack of diversity and inclusivity in the healthcare workforce. Black and Latinx doctors comprise 5% and 5.8% of all active US physicians, respectively, while 13.4% and 18.5% of the general American population are Black and Latinx.^17^ Considering that, in the United States, 7.5% of non-Hispanic White, 11.7% of Black, 12.5% of Hispanic, 9.2% of Asian Americans have diabetes, gaps in healthcare workforce representation may explain disparities in patient-reported cultural competency measures.^15^ Moreover, racial minority groups are less likely to have a usual source of care and are more likely to use the emergency department as a usual source of care.^19^, ^20^, ^21^

Similarly, the number of physicians trained to give LGBTQ-competent health care is low overall.^22^ A survey of several academic institutions in 2015 revealed that 52% had no established training programs for LGBTQ health. Majority of the institutions who offered said training programs were located in areas with an already established LGBTQ health care center.^22^ While the development of ethnic and sexual identities occurs concurrently, both identities are highly independent of each other and have equal contributions to a person's sense of self and community.^23^ Therefore, it is possible that despite sharing an ethnic identity with their provider, LGBTQ patients may still be unable to identify with them.

Although migrant representation is high in the US healthcare workforce, in which 29% of currently active US physicians are foreign-born, there is a disproportionate distribution of international medical graduates (IMGs) across US specialties, with the majority working as internists, paediatricians, neurologists, psychiatrists, and family practitioners.^24^^,^^25^ In 2015, out of all graduate medical trainees in other specialties involved in diabetes care, only 2% of orthopaedic surgery trainees, 5% of ophthalmology trainees, and 6% of emergency medicine trainees were IMGs.^24^^,^^25^

#### Cultural humility

Our findings suggest that Asian respondents had lower odds of feeling respected by their providers. While the point estimate demonstrates lower odds among Latinx respondents to report frequently being treated with respect by providers, this finding was not statistically significant (Figure. 1). Older respondents had decreased odds of reporting being frequently treated with respect by their providers. Black individuals and those not part of the workforce had greater odds of reporting being asked about their opinions or beliefs in their care (Figure. 1). While these latter positive associations are promising, the influence of acquiescence survey bias, in which respondents report what they believe to be the favorable instead of the true answer to questions, cannot be ruled out.^26^ Therefore, these findings should be verified in future studies.

Although the supplemental questions funded by the Office of Minority Health were categorised as measures of cultural competency, questions pertaining to the frequency of being treated with respect and asked about opinions or beliefs in care by providers overlap with concepts of cultural humility. In contrast to cultural competency, which is focused on gaining the necessary knowledge and skill to care for CALD populations, cultural humility involves provider self-evaluation and self-critique in the purview of addressing power imbalances in the patient-provider relationship.^27^ That is, cultural humility highlights provider introspection and openness to cultivate honest and respectful patient-provider interactions.^27^

Asian Americans are often viewed in the lens of the model minority myth - smart, successful, and accomplished as a whole.^28^ However, despite being viewed as a single entity, there is significant diversity within this group, culturally and socioeconomically.^29^ For example, disaggregated AANHPI data suggest that Filipino Americans were two times more likely than non-Hispanic white people to have diabetes, a disparity that was not as severe among other AANHPI groups. The aggregation of this population in much of biomedical research, combined with the endorsement of the model minority stereotype, has deleterious impacts on this population's health.^4^^,^^30^, ^31^, ^32^ Asians have the highest undiagnosed rate of diabetes across ethnicities in the USA, and they are also one the most underscreened populations.^33^ Despite having a higher risk to develop diabetes, Asian Americans were less likely to be managed in accordance with recommended guidelines.^33^^,^^34^ Our findings further underscore the importance of cultural humility in the care of cultural minorities with diabetes, particularly Asian Americans.

#### Effective communication, language barriers, and health literacy

Non-English speakers had lower odds of reporting receiving easy-to-understand information (Figure. 2). In contrast, respondents from higher income households had greater odds of reporting receiving information about their care that they easily understood.

This disparity may be due to higher health literacy rates among higher income households and the lack of linguistically appropriate services provided for non-English speaking populations.^35^^,^^36^ In the United States, non-English speakers have higher rates of diabetes and food insecurity.^6^ Overall, language barriers had negative impacts on patient and provider satisfaction, adverse patient outcomes, and patient safety.^37^ Limited English proficiency (LEP) is linked to lower health literacy and amplifies the negative health outcomes from lower income and lack of health insurance.^38^ Proper patient communication includes the provision of language assistive services and easy-to-understand information, aims to address language barriers and gaps in health literacy, and is a key component of culturally competent care.^12^ Past studies on language-concordant care showed improvement in patient outcomes such as glycaemic control.^39^

This issue of communication is not limited to those with LEP. Minorities as a whole face significant challenges in healthcare communication, and race discordance has been associated with poorer physician-patient communication.^40^^,^^41^ Despite this, interventions to improve health care provider communication skills alone have limited benefit.^42^ Other methods to address this issue may prove to be more effective upon further study.

#### Clinical implications and actionable points

In addition to the clinical implications of differences in genetics and nonmodifiable risk factors, cultural minority groups are at an increased risk for worse outcomes in diabetes due to structural racism, chronic exposure to psychosocial stress, lack of healthcare coverage and self-monitoring, food insecurity, and language barriers.^5^^,^^6^^,^^18^^,^^43^ Therefore, tailoring evidence-based interventions to the social and cultural contexts of patients may improve patient self-management and reduce disparities among diabetics.^6^

While there have been innumerable efforts to incorporate cultural competency in medical education nationwide, research on the contemporary state of cultural competency in healthcare services on a large and nationally representative sample is lacking.^7^ Furthermore, due to the lack of granular information collected, most studies on cultural competency in healthcare, including this one, stratify patient populations only into the largest racial and ethnic groups: White, Black, Hispanic, and Asian.^5^^,^^7^ Other groups such as American Indians, Alaskan Natives, Pacific Islanders, people of mixed races are remarkably heterogeneous but are pooled into a group of “Other” races.^5^ We therefore recommend future studies to make efforts to attain more granular information not only on patients’ racial and ethnic background, but also their sexual orientation, gender identity and expression (SOGIE), urban-rural household classification, languages spoken, country of origin, and the intersectionality of all these variables.^5^

Future efforts to promote cultural competency in diabetes care and reduce healthcare disparities may learn from past studies that have demonstrated improvements in clinical outcomes among certain populations who received culturally tailored interventions.^6^, ^7^, ^8^, ^9^, ^10^ Cultural competency and cultural humility should continue to be taught in medical education for all healthcare workers and ancillary staff. Increasing representation in the healthcare workforce and racial/ethnic, language, and gender concordance may improve patient-provider interactions and clinical outcomes.^44^ System-wide interventions are needed not only to integrate cultural and linguistic diversity in care, but also to address other determinants of healthcare disparities such as health coverage, poverty, employment, education, and literacy.^6^^,^^9^^,^^10^

#### Limitations

This study is limited by the self-reported nature of a diabetes diagnosis and the lack of information on the classification and severity of diabetes (type I vs. type II, insulin requiring or non-insulin requiring). In addition, the five supplemental questions of the NHIS 2017 do not fully encompass the domains of cultural competency, cultural humility, and patient-provider communication. Future work involving mixed and qualitative methods (e.g. focused group discussions) are necessary to elucidate the complex interplay between the provision of culturally competent care and clinical outcomes among patients with diabetes.

Although the authors attempted to analyze as many identity groups as possible, there is limited granularity of collected information on respondents’ racial/ethnic background, sexual orientation, socioeconomic status, and other sociodemographic information. It is entirely possible that other aspects of an individual's culture – such as religion or spirituality – contribute to culture in and preference for culturally competent care in ways that we are unable to capture in the NHIS. The inclusion of a broad range of variables cognizant of the possibility of some degree of collinearity among them was intentionally done to identify those that are most strongly associated with the desire for – and access to – culturally competent care. We look forward to studies that further disentangle these complex and interrelated factors.

Moreover, racial minorities are less likely to have a usual source of care and are more likely to use the emergency department as a usual source of care; prior studies found that Black patients with diabetes had fewer primary care visits and more emergency department visits than white patients with diabetes. The variable nature of their care likely influences their view on provider cultural competency.^20^^,^^21^^,^^45^ However, no data is available on the usual sources of care of the respondents.

Furthermore, race/ethnicity classifications do not account for people who identify with categories not included in the NHIS, an inherent limitation of these classifications more broadly. Additionally, small sample size precluded a more granular assessment of disaggregated subgroups such as for AANHPI. This study is also limited by response biases inherent to a survey design, given the survey's retrospective nature. Lastly, healthcare provider information was not supplied and therefore patient-provider concordance and the purpose of the patient-provider interaction cannot be determined.

### Contributors

JAP and JMG contributed equally to this manuscript. All authors were involved in the writing, editing, and review of the original draft. In addition, JAP was responsible for conceptualisation, data curation, formal analysis, investigation, methodology, project administration, and validation. JMG was responsible for methodology, project administration, resources, software, supervision, validation, and visualisation. JSY was involved in the formal analysis, investigation, and methodology. MAE was involved in the methodology and visualisation. ECD was involved in the conceptualisation, data curation, formal analysis, methodology, software, and visualisation. MGY was involved in the conceptualisation, data curation, investigation, methodology, project administration, supervision, validation, and visualization of the project.

### Funding

No funding was used in the preparation of this work.

### Data sharing statement

All data used in this study can be accessed at https://www.ipums.org/healthsurveys.shtml.

## Declaration of Interests

All authors declare no conflicts of interest. No funding was involved in this research.
